# Visualization system to identify structurally vulnerable links in OHT railway network in semiconductor FAB using betweenness centrality

**DOI:** 10.1371/journal.pone.0307059

**Published:** 2024-07-12

**Authors:** Jinwoo Choi, Youngbin Park, Yeeun Choi, Sehyeon Kim, Heewon Lee, Hyunwoo Park

**Affiliations:** 1 Graduated School of Data Science, Seoul National University, Seoul, South Korea; 2 Samsung Electronics Co., Ltd., Giehung Campus, Giheung-gu, Yongin-si, Gyeonggi-do, Republic of Korea; Borgwarner, GERMANY

## Abstract

In semiconductor fabrication (FAB), wafers are placed into carriers known as Front Opening Unified Pods (FOUPs), transported by the Overhead Hoist Transport (OHT). The OHT, a type of Automated Guided Vehicle (AGV), moves along a fixed railway network in the FAB. The routes of OHTs on the railway network are typically determined by a Single Source Shortest Path (SSSP) algorithm such as Dijkstra’s. However, the presence of hundreds of operating OHTs often leads to path interruptions, causing congestion or deadlocks that ultimately diminish the overall productivity of the FAB. This research focused on identifying structurally vulnerable links within the OHT railway network in semiconductor FAB and developing a visualization system for enhanced on-site decision-making. We employed betweenness centrality as a quantitative index to evaluate the structural vulnerability of the OHT railway network. Also, to accommodate the unique hierarchical node-port structure of this network, we modified the traditional Brandes algorithm, a widely-used method for calculating betweenness centrality. Our modification of the Brandes algorithm integrated node-port characteristics without increasing computation time while incorporating parallelization to reduce computation time further and improve usability. Ultimately, we developed an end-to-end web-based visualization system that enables users to perform betweenness centrality calculations on specific OHT railway layouts using our algorithm and view the results through a web interface. We validated our approach by comparing our results with historically vulnerable links provided by Samsung Electronics. The study had two main outcomes: the development of a new betweenness centrality calculation algorithm considering the node-port structure and the creation of a visualization system. The study demonstrated that the node-port structure betweenness centrality effectively identified vulnerable links in the OHT railway network. Presenting these findings through a visualization system greatly enhanced their practical applicability and relevance.

## Introduction

In the complex process of semiconductor chip fabrication, which includes hundreds to thousands of steps such as lithography and deposition, wafers—the silicon substrate on which chips are fabricated—are placed into carriers known as Front Opening Unified Pods (FOUPs). Typically, a group of 25 wafers, referred to as a LOT, is transported by these FOUPs. Overhead Hoist Transport (OHT), a type of Automated Guided Vehicle (AGV), facilitates the movement of FOUPs between machines to carry out these processes. Controlled by the Automated Material Handling Systems (AMHSs), these OHTs, numbering in the hundreds within a FAB, travel an average of 20 kilometers per day [1].

The OHT railway network is characterized by its unidirectional flow and has only two types of intersections: two incoming-one outgoing, and one incoming-two outgoing. Moreover, the maximum velocity constraint varies depending on the rail type, with curved rails having a lower maximum velocity than straight rails [2]. These limited intersections and rail constraints frequently contribute to potential congestion in the OHT railway network.

Furthermore, OHT routes are primarily determined by Single Source Shortest Path (SSSP) algorithms, such as Dijkstra’s [3, 4]. This routing method can lead to certain nodes and links becoming overloaded with multiple routes, thereby inducing congestion. Although there are other factors involved in determining OHT routes and rerouting algorithms are employed during congestion, these measures are not fully effective in preventing congestion.

For these reasons, frequent congestion and deadlocks in the FAB lead to diminished productivity and, by extension, reduce company revenue. Therefore, maintaining a consistent and normal operation of the OHT transportation system is crucial for the profitability and efficiency of semiconductor companies. Identifying structurally vulnerable links in railway networks and applying targeted strategies in advance to address these vulnerabilities can significantly lessen congestion. It facilitates the anticipation and control of potential congestion points, improving network management [5]. Recognizing and remedying a network’s vulnerable links can optimize traffic flow [6], boost network reliability, and reduce the effects of disruptions, thereby enhancing network connectivity and reliability [7], ultimately reducing congestion.

In the field of OHT railway networks, the identification of vulnerable links has traditionally been heavily reliant on the experience of domain experts and historical data. While domain experts’ long-term experience and historical data on a specific railway layout can be effective, this approach has limitations. The variability in railway layout structures and process flows makes it challenging to rely solely on historical data and expert experience to assess the vulnerability of links. For instance, the railway layout can be changed due to the placement or removal of equipment, such as fabrication tools and stockers for idle FOUPs. Furthermore, the fabrication process may undergo modifications, including the addition or omission of steps, leading to deviations in historical patterns. Moreover, historical data and experience are inadequate for newly constructed OHT networks where past trends do not exist. Consequently, the traditional method of detecting vulnerable links, based on the judgment of domain experts and historical data, becomes less reliable. Therefore, a more systematic and quantitative approach to measuring the vulnerability of links is necessary. This will improve the reliability of vulnerable link detection in OHT railway networks despite these variations and better support domain experts in their decision-making process.

This study focused on identifying structurally vulnerable links in the OHT railway network within semiconductor FAB and developing a visualization system to aid on-site decision-making based on these findings. The academic and practical contributions are divided into the development of an algorithm and the creation of a visualization system.

Firstly, we employed betweenness centrality to evaluate the structural vulnerability of the OHT railway network, considering the node-port structure and developed a new betweenness centrality calculation algorithm. Betweenness centrality has been widely recognized for its effectiveness in identifying critical points within a network in graph theory. It serves as a quantitative identifier to detect vulnerable links but has not been practically applied to the OHT railway network before. This study is the first to apply this concept in the OHT railway network field, detecting vulnerable links while reflecting the characteristics of the OHT railway network compared to other networks by considering the node-port structure, making the situation in the FAB more realistically represented. This enhanced the existing Brandes algorithm to be more realistic and practical, maintaining the same time complexity as the node-only structure algorithm and adding parallelization capabilities, thus drastically reducing computation time and increasing practical usability on-site. Depending on the number of processors used simultaneously in computation, we confirmed a 5.7-times improvement in computational speed (from 442 seconds to 78 seconds).

Secondly, based on the results of the betweenness centrality calculation considering the node-port structure, we developed a web-based visualization system to visualize and identify these vulnerable links in the OHT railway. We represented the OHT railway as a graph structure and calculated the betweenness centrality of each node and link based on the modified Brandes algorithm. The vulnerability calculation results were visually implemented through the graph to allow users to intuitively recognize vulnerable sections, and a CSV file summarizing the results was provided to facilitate further data analysis and use.

We validated the reliability of our research findings by comparing the vulnerable links detected using our modified betweenness centrality calculation algorithm considering the node-port hierarchy against historically vulnerable links in the same OHT railway network provided by Samsung Electronics. Our study demonstrated that the node-port structure betweenness centrality can effectively identify structurally vulnerable links in the OHT railway network, and providing these effective vulnerable link identification results through a web-based visualization system significantly enhances the practical applicability and relevance of our research.

In this paper, we will use the term ‘link’ instead of ‘edge’, as ‘link’ is more commonly used within the domain of the OHT railway network. In the related work section, we will briefly introduce some existing research and concepts related to visualization systems for the OHT railway network, betweenness centrality, and vulnerability. Following this, the materials and methods section will detail the data utilized, our visualization system, and our modified algorithm. Subsequently, we will present a result using real data of the OHT railway network provided by Samsung Electronics and discuss these findings.

## Related works

### Visualization system for OHT railway network

Research on the OHT (Overhead Hoist Transport) railway network has predominantly centered around routing algorithms [8–11], dispatching [1, 12–15], design [16, 17], and fault detection [18]. In contrast, few studies have addressed monitoring or visualization systems for the OHT railway network, particularly concerning the vulnerability of links. Despite the importance of evaluating and monitoring vulnerable links for improving network reliability and management, research on quantitatively identifying vulnerable links in the OHT railway network has not advanced significantly relative to its necessity. Aside from the study conducted by Lee et al. [2] in the following section, no additional research has been undertaken, as far as we are aware.

In 2018, S. Lee et al. developed a Congestion Monitoring System (CMS) and explored various congestion metrics, proposing new rerouting algorithms based on these metrics. Their research primarily addressed sections, typically defined as groups of links between intersections, rather than individual links. They not only measured basic metrics such as traffic volume and average speed for each section but also introduced new metrics: the Absolute Congestion Index (ACI) and the Relative Congestion Index (RCI). The ACI, calculated as the average speed of moving OHT divided by the maximum speed at a section, identifies structurally congested sections. On the contrary, the RCI, using a moving average of OHT speed over fixed periods, detects instant congestion since it reflects the typical traffic conditions of a section. These metrics are assessed section-wise every 10 minutes and visualized on the CMS, enabling real-time monitoring by users.

Furthermore, they proposed a new rerouting algorithm, Congestion-Based Routing (CBR), which incorporated ACI as a penalty term to the original Distance-Based Routing (DBR) cost, accounting for dynamic congestion scenarios. The performance of CBR was validated through simulations designed by the researchers. However, it is important to note that their system primarily reflects the current condition of the OHT railway and does not provide a fixed index for ranking structurally vulnerable links in the network.

While their research effectively monitored congestion, continuous monitoring of the CMS was necessary to identify congestion sections because metrics like ACI and RCI were based on dynamic conditions, meaning they change according to the current state of the OHT railway. In other words, their system did not offer a fixed index to determine or rank structurally vulnerable links, though it can reflect the current congestion situation in real-time.

As can be seen from the preceding discussion, the field of quantitatively assessing the vulnerability of the OHT railway network is notably under-researched, with the existing study requiring continual monitoring by operators and lacking the capability to identify vulnerabilities when network structures change. Recognizing these limitations, our research aimed to quantitatively ascertain the inherent structural vulnerabilities using only the network structure. We employed betweenness centrality as a fixed index to assess vulnerability, enabling this approach. In this process, we developed an algorithm that considers the unique node-port structure of the OHT railway network, ensuring a realistic representation of the structure. Subsequently, we developed a visualization system to make the results of vulnerability assessment practically usable. This development is significant as it provides semiconductor industry domain experts with visual information about vulnerable links, enabling them to make more effective management decisions regarding the network.

### Betweenness centrality

Betweenness centrality, a popular index in graph analysis, was initially introduced by Freeman (1977) [19] and Anthonisse (1971) [20]. It measures the connectivity of nodes or links within a network and has historically been used to identify critical bridges or bottleneck links in various network structures.

The Brandes algorithm, proposed by Ulrik Brandes in 2001, is the most commonly employed method for calculating betweenness centrality. It calculates centrality scores by accumulating them from the farthest node to the source node following the predecessors. This algorithm improved time and space efficiency over previous methods [21].

However, the Brandes algorithm faces challenges with large-scale graphs due to its computational intensity. This limitation has prompted the development of various modified algorithms that incorporate approximation methods and parallel computation strategies to reduce computation time while retaining the fundamental idea of the Brandes algorithm [22]. Additionally, research has been conducted on updating betweenness centrality in dynamic graphs without requiring full recalculations for each graph update. These update algorithms vary depending on the type of update, such as additions or deletions of nodes/links and weight changes.

Specifically, Nasre et al. (2013) introduced an incremental algorithm updating betweenness centrality scores in a graph when a new link is added or an existing link’s weight is reduced [23]. Similarly, Bhandari et al. (2017) developed methods to efficiently update betweenness centrality in dynamic networks with additions or deletions of links or nodes [24]. In addition, Khopkar et al. (2014) targeted social networks and devised fast, incremental update algorithms for various network metrics, including betweenness centrality [25]. Further enhancing these advancements, Feng et al. (2022) and González et al. (2022) introduced more efficient approaches using parallel computing for large-scale network analysis and improved the scalability of the Brandes algorithm, respectively [26, 27].

### Vulnerability

Vulnerability is a concept broadly applied across various domains such as transportation networks (including roads, railways, and airways), supply chains, and even graph neural network models. Vulnerability can be applied to any structure that can be modeled as a graph with nodes and links.

For our study, we adopt Berdica’s definition of vulnerability, which originally pertains to road networks as “susceptibility to incidents that can result in considerable reductions in road network serviceability.” We adapt this definition to the OHT railway network by replacing ‘road network’ with ‘OHT railway network’ and ‘incidents’ with ‘congestion’ [28].

According to the survey paper of S. Pan et al., vulnerability analysis can be categorized into four areas: traditional topology analysis, model optimization, simulation, and data-based approaches, noting that these methods can overlap depending on the research focus and methodology [29]. Topological analysis relies on graph topology and static conditions, utilizing metrics like average distance, global and local efficiency, and centrality scores such as betweenness centrality to identify vulnerabilities.

In contrast, simulation and data-based approaches accommodate both static and dynamic conditions. Simulations often include attack methods that examine the impact of node or link removals on network indices such as betweenness centrality. This attack can be conducted either randomly or according to a specific strategy. Li et al. (2019) combined simulation methods with network topology analysis to assess the robustness of air transport networks under both random failures and targeted attacks [29, 30]. Data-based approaches utilize real data to consider historical trends or real-time conditions.

A noteworthy study by Furno (2018) presented a framework to identify vulnerabilities in extensive road networks while employing a graph corresponding to the dynamic road networks and examined the consequences of eliminating nodes on the functionality and efficiency of multi-modal transportation networks. [31]. Another data-driven study by Zhou et al. (2019) utilized real route data to develop a new efficiency metric for assessing air transport network robustness [32].

Our research aligns with topological analysis, selecting betweenness centrality as a suitable metric due to its relevance in analyzing the unique node-port structure of the OHT railway network where routes are determined by a SSSP algorithm. This choice not only underscores the applicability of betweenness centrality in static condition analysis but also paves the way for extending our study to include attack-based and dynamic condition analyses.

To the best of our knowledge, this is the first research to apply betweenness centrality for identifying and visualizing structurally vulnerable links within the OHT railway network. Furthermore, our study took into account the realistic node-port structure in the FAB and utilized a modified Brandes algorithm tailored to the hierarchical structure. This modification enhanced the practicality of our algorithm, potentially extending its application to other similarly structured systems.

## Materials and methods

### Data and notation

In this study, we used node, link, and port data in the real FAB provided by Samsung Electronics. However, due to the sensitive nature of the information, certain specific details were either preprocessed or not disclosed to maintain confidentiality. For example, the distances of links were normalized, and the exact coordinates of each node and port were not revealed by the company, reflecting their proprietary and confidential status.

The features for each dataset are summarized in Table 1. Each node, link, and port were assigned unique IDs, referred to as Encoded_Id in the ‘Node’ and ‘Link’ datasets, and Encoded_Port in the ‘Port’ dataset. The ‘Link’ data included the IDs of the source and target nodes that correspond in the ‘Node’ dataset (Encoded_From_Node, Encoded_To_Node), as well as normalized encoded distances (Encoded_Distance). In the ‘Port’ dataset, the Encoded_Id feature represents the ID of the nearest node to which it is mapped.

**Table 1:** Features of datasets.

Dataset	Features
Node	Encoded_Id
Link	Encoded_Id, Encoded_From_Node, Encoded_To_Node, Encoded_Distance
Port	Encoded_Port, Encoded_Id

We selected a particular layout for our research due to its size, as it was the largest among the layouts offered by Samsung Electronics. Additionally, it included crucial structural components, such as bridges that connect different areas.

Table 2 below summarizes the size of each node, link, and port data, along with additional statistics pertaining to the OHT railway layout data we utilized.

**Table 2:** Statistics of data.

Statistics	Value
Count of Nodes	15911
Count of Links	18204
Count of Ports	40325
Average Distance	0.4398
Max. number of ports mapped to a node	22
Min. number of ports mapped to a node	0
Avg. number of ports mapped to a node	2.53
Std. number of ports mapped to a node	4.23

**Fig 1:**
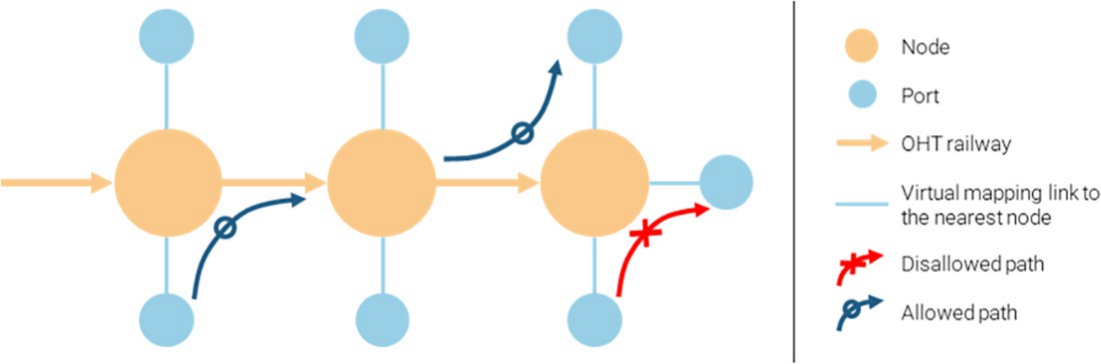
Node-port structure of OHT railway. Nodes are represented by orange circles, and ports are represented by blue circles. Each port is assigned to its nearest node. Paths between ports in different nodes are allowed, while port-to-port paths within the same node are disallowed.

To further explain the node-port structure in the OHT railway network, nodes do not represent the actual source or target points but serve as sensors that record the positions and velocities of the nearest OHTs. In contrast, ports are the actual stopping points where FOUPs are loaded and unloaded. The conceptual figure depicted in Fig 1 illustrates the node-port structure. In Fig 1, the large orange circles represent nodes, while the smaller blue circles represent ports. Ports are assigned to their nearest node, with each node accommodating multiple ports. The actual travel path was assumed to be from port to port, rather than from node to node, as shown by the navy arrows labeled ‘Allowed path’ in Fig 1. Impractical port-to-port paths within the same node were disregarded, represented by the red arrows labeled ‘Disallowed path.’ Considering the unique structure of the OHT railway network, we developed a new algorithm to calculate betweenness centrality.

We have detailed our modified algorithm and its corresponding proof in the subsequent sections. For this purpose, we utilized a range of symbols, with their respective summaries presented in Table 3 below.

**Table 3:** Summary of notation.

Symbol	Description
*V*	The set of nodes
*E*	The set of links
*G* = (*V*, *E*)	The graph with node *V* and link *E*
*BC*(*v*)	Betweenness centrality of *v*
*BC*_*p*_(*v*)	Betweenness centrality of *v* in node-port structure
*σ*_*st*_	The number of shortest paths from node *s* to node *t*
*σ*_*st*_(*v*)	The number of shortest paths from node *s* to node *t* passing the node *v*
*σ*_*sw*_(*v*, {*v*, *w*})	The number of shortest paths from node *s* to node *t* passing the node *v* and link (*v*, *w*)
*δ*_*st*_(*v*)	Pair dependency from node *s* to node *t* passing the node *v*
*δ*_*st*,*p*_(*v*)	Modified pair dependency from node *s* to node *t* passing the node *v* in node-port structure
*δ*_*s*•,*p*_(*v*)	sum of all modified pair dependencies from source *s* to all the target
*p*(*v*)	The number of ports in node *v*
*S*	The sequence of successors from a source
*P*	The set of predecessors of a node from a source

Since the data we used is confidential FAB data from Samsung Electronics and cannot be disclosed, we have provided our code and OHT railway layout open data from SMAT 2022 [33] on our GitHub repository instead. The result obtained using this SMAT 2022 layout is also included in S1 Figure in S1 File.

### Visualization method

Since the coordinates of the nodes were confidential, we used a force-directed layout algorithm to visualize the OHT railway network and set a strong stiffness constant to maintain distance as much as possible. However, due to the complexity of the network, with its numerous nodes and links, the resulting graph often appeared dense and hairball-like. To improve clarity, we integrated a grid-force into the algorithm to align the nodes to the nearest grid points. The grid size, a predefined hyperparameter, resulted in a more rectangular graph layout, approximating the actual OHT railway layout. To implement the visualization system as a web application, we used Flask, a Python-based web framework, for the backend and MongoDB as the database platform. A detailed explanation of our visualization system is provided in the ‘Results and discussion’ section.

### Betweenness centrality calculation algorithm

For the node-only structure, we used the Brandes algorithm to calculate betweenness centrality using the Python NetworkX library.

To account for the node-port structure depicted in Fig 1, we modified the Brandes algorithm, ensuring that its time complexity remains equivalent to that of a node-only structure.

The definition of betweenness centrality is shown in Eq (1).

$$\begin{array}{c} BC(v)=\sum_{s\neq v\neq t\in V}\frac{\sigma_{st}\left(v\right)}{\sigma_{st}}=\sum_{s\neq v\neq t\in V}\delta_{st}\left(v\right) \end{array}$$ (1)

In this equation, $\delta_{st}\left(v\right)=\frac{\sigma_{st}\left(v\right)}{\sigma_{st}}$ is known as the pair dependency. Betweenness centrality is the sum of all pair dependencies passing through a given node or link.

$$\begin{array}{c} BC_{p}\left(v\right)=\sum_{s\neq v\neq t\in V}\frac{\sigma_{st}\left(v\right)}{\sigma_{st}}p\left(s\right)p\left(t\right)=\sum_{s\neq v\neq t\in V}\delta_{st,p}\left(v\right) \end{array}$$ (2)

To incorporate the node-port structure, we modified the conventional definition of betweenness centrality, as provided in Eq (2). Here, p(s) and p(t) denote the number of ports at the source and target nodes, respectively. Using the standard Brandes algorithm while treating ports as additional nodes would increase time complexity proportional to the rise in nodes and links. To address this issue, we adjusted the equation and introduced a modified pair dependency term, defined as follows:

$$\delta_{st,p}\left(v\right)=\frac{\sigma_{st}\left(v\right)}{\sigma_{st}}p\left(s\right)p\left(t\right)$$

$$\begin{array}{c} \delta_{s•,p}\left(v\right)=\sum_{t\in V}\delta_{st,p}\left(v\right)=\sum_{t\in V}\sum_{w:v\in P_{s}\left(w\right)}\delta_{st,p}\left(v,\left\{v,w\right\}\right)=\sum_{w:v\in P_{s}\left(w\right)}\sum_{t\in V}\delta_{st,p}\left(v,\left\{v,w\right\}\right) \end{array}$$ (3)

To compute the sum of modified pair dependencies, we followed the original Brandes algorithm proof steps. In Eq (3), *δ*_*s*•,*p*_(*v*) is the sum of all modified pair dependencies from source *s* to all the targets. *δ*_*st*,*p*_(*v*, {*v*, *w*}) represents the modified pair dependency passing through node *v* and link (*v*, *w*). *P*_*s*_(*w*) denotes the set of predecessors of *w*.

$$\begin{array}{c} \delta_{st,p}\left(v,\left\{v,w\right\}\right)=\{\begin{array}{cc} \frac{\sigma_{sv}}{\sigma_{sw}}\cdot p\left(s\right)p\left(w\right) & \text{if}\;t=w\\ \frac{\sigma_{sw}\left(v,\left\{v,w\right\}\right)\cdot \sigma_{wt}}{\sigma_{st}}\cdot p\left(s\right)p\left(t\right)=\frac{\sigma_{sv}}{\sigma_{sw}}\cdot \frac{\sigma_{st}\left(w\right)}{\sigma_{st}}\cdot p\left(s\right)p\left(t\right) & \text{if}\;t\neq w \end{array} \end{array}$$ (4)

*δ*_*st*,*p*_(*v*, {*v*, *w*}) can be expressed as two terms, as shown in Eq (4). If *t* = *w*, then it simply becomes $\frac{\sigma_{sv}}{\sigma_{sw}}\cdot p\left(s\right)p\left(w\right)$ since *σ*_*sw*_(*v*, {*v*, *w*}) = *σ*_*sv*_. In the case of *t* ≠ *w*, *σ*_*st*_(*v*, {*v*, *w*}) = *σ*_*sw*_(*v*, {*v*, *w*}) ⋅ *σ*_*wt*_ and because *σ*_*st*_(*w*) = *σ*_*sw*_ ⋅ *σ*_*wt*_, *σ*_*wt*_ becomes $\frac{\sigma_{st}\left(w\right)}{\sigma_{sw}}$. So the modified pair dependency term becomes $\frac{\sigma_{sv}}{\sigma_{sw}}\cdot \frac{\sigma_{st}\left(w\right)}{\sigma_{st}}\cdot p\left(s\right)p\left(t\right)$ when *t* ≠ *w*.

$$\begin{array}{cc} \sum_{w:v\in P_{s}\left(w\right)}\sum_{t\in V}\delta_{st,p}\left(v,\left\{v,w\right\}\right) & =\sum_{w:v\in P_{s}\left(w\right)}(\frac{\sigma_{sv}}{\sigma_{sw}}\cdot p\left(s\right)p\left(w\right)+\sum_{t\in V\backslash \{w\}}\frac{\sigma_{sv}}{\sigma_{sw}}\cdot \frac{\sigma_{st}\left(w\right)}{\sigma_{st}}\cdot p\left(s\right)p\left(t\right))\\ & =\sum_{w:v\in P_{s}\left(w\right)}\frac{\sigma_{sv}}{\sigma_{sw}}\cdot (p\left(s\right)p\left(w\right)+\delta_{s•,p}\left(w\right)) \end{array}$$ (5)

By incorporating this formula into the original sum expression, it is represented as illustrated in the aforementioned Eq (5), and the last term is *δ*_*s*•, *p*_(*w*). As a result, we obtained an accumulation similar to that of the Brandes algorithm.

**Algorithm 1** Calculation of betweenness centrality

**Input**: *G* = (*V*, *E*), *weight*, *port*, *nodes*

**Output**: *BC*_*node*(*v*), *BC*_*link*(*e*) for all *v* ∈ *V* and *e* ∈ *E*

1: **for** *v* ∈ *V* **do**

2:  *BC*(*v*) ← 0

3: **end for**

4: **for** *e* ∈ *V* **do**

5:  *BC*(*e*) ←0

6: **end for**

7: **if** *nodes* = None **then**

8:  *nodes* = *V*

9: **end if**

10: **for** *s* ∈ *nodes* **do**

11:  **if** *weight* = None **then**

12:   *S*, *P*, *σ* = *SSSP*(*G*, *s*)

13:  **else**

14:   *S*, *P*, *σ* = *SSSP*(*G*, *s*, *weight*)

15:  **end if**

16:  **if** *port* = None **then**

17:   *BC* = *BA*(*BC*, *S*, *P*, *sigma*, *s*)

18:  **else**

19:   *BC* = *BA*_*p*_(*BC*, *S*, *P*, *sigma*, *s*, *port*)

20:  **end if**

21: **end for**

22: *BC*_*node* = {}

23: **for** *s* ∈ *S*(*v*) **do**

24:  pop *BC*_*s*_ from *BC*(*s*)

25:  *BC*_*node*(*s*) ← *BC*_*s*_

26: **end for**

27: *BC*_*link* = *BC*

28: **return** *BC*_*node*(*v*), *BC*_*link*(*e*) for all *v* ∈ *V* and *e* ∈ *E*

The pseudocode for computing betweenness centrality for both nodes and links is illustrated in Algorithm 1. The algorithm’s inputs include the graph *G*, selected features for weight, port data, and nodes for parallelization later. In the initial setup, lines 1 to 6, the betweenness centrality values for each node and link in *G* are initialized to zero. In the next segment, lines 7 to 9, the algorithm manages cases without employing parallelization. If a specific set of nodes is not provided, the algorithm defaults to utilizing all nodes within *G*. Subsequently, the algorithm proceeds to compute the SSSP for a node *s*. If no weight parameter is provided, it employs Breadth-First Search (BFS). In contrast, for weighted directed graphs, Dijkstra’s algorithm is utilized instead. Both methodologies yield three key outputs: *S*, *P*, and *σ*. Here, *S* represents a sequence of nodes commencing from the source *s* to all target nodes, ordered by proximity. *P* is a record of predecessors for each node within *S*, and *σ* is the total count of shortest paths originating from source *s* to each target node *t*.

Subsequently, the algorithm checks if port data is available. If not, the original Brandes algorithm is applied for betweenness centrality calculations. If port data is available, the algorithm employs our modified Brandes algorithm as described above.

In the final stages, encompassed in lines 22 to 27, the algorithm separates the betweenness centrality calculations for nodes and links, ultimately yielding both values.

**Algorithm 2** *BA*_*p*_: Accumulation of modified pair-dependencies

**Input**: *BC*, *S*, *P*, *σ*, *s*, *port*

**Output**: *BC*(*x*) for all *x* ∈ *V* ∪ *E*

1: **for** *v* ∈ *S* **do**

2:  *δ*(*v*) ←0

3:  **if** *v* != *s* **then**

4:   *sum*_*ports* += *port*(*v*)

5:  **end if**

6: **end for**

7: *BC*(*s*) += *port*(*s*) * *sum*_*ports*

8: **while** *S* **do**

9:  pop *w* from *S*

10:  *coeff* = (*port*(*s*) * *port*(*w*) + *δ*(*w*))/*σ*(*w*)

11:  **for** *v* ∈ *P*(*w*) **do**

12:   *c* = *σ*(*v*) * *coeff*

13:   *BC*({*v*, *w*}) += *c*

14:   *δ*(*v*) += *c*

15:  **end for**

16:  **if** *w*! = *s* **then**

17:   *BC*(*w*) += *δ*(*w*) + *port*(*s*) * *port*(*w*)

18:  **end if**

19: **end while**

20: **return** *BC*(*x*) for all *x* ∈ *V* ∪ *E*

Algorithm 2 presents our modified Brandes algorithm. The inputs for this algorithm include betweenness centrality *BC*, sequences *S*, predecessors *P*, the count of shortest paths *σ*, source node *s*, and port data *port*. Initially, every *δ* term is set to zero.

The algorithm then computes the cumulative sum of the fraction of the number of ports to its *σ* of all target nodes *v* from source *s*, excluding the source node *s* itself. This step incorporates the betweenness centrality of the source, considering that the actual starting point is the ports in *s*, rather than the node *s* itself. Therefore, in line 7, the betweenness centrality for source *s* is calculated by multiplying the number of ports in *s* with the *sum*_*source*_*dependency* term.

From lines 8 to 19, the algorithm proceeds with the accumulation of modified pair dependencies, working backward from the most distant target node to the source node. The main difference from the standard Brandes algorithm is in the coefficient, specifically *port*(*s*) * *port*(*w*). In line 17, the factor *port*(*s*)*port(*w*) is also considered to account for the target node *t*, acknowledging that the actual targets are the ports within *t*, similar to the source node case above.

This algorithm enables the calculation of modified pair dependencies, taking into account port, from the source node *s*. By iterating this process over all nodes *s* in the set *V*, it is possible to accurately determine the betweenness centrality for both nodes and links in the network.

Along with betweenness centrality, we also calculated stress centrality as a supplementary indicator of structural vulnerability. Stress centrality is the sum of the number of shortest paths passing through a node or link, while betweenness centrality is the sum of pair-dependencies. Both can be calculated for node and node-port structures. Detailed information about stress centrality is provided in S1 Appendix in S1 File, and algorithms for its calculation are given in S1 and S2 Algorithms in S1 File.

### Parallelization

In our effort to optimize computational efficiency, we employed a modified accumulation algorithm which notably reduced time complexity. However, for an average OHT railway network graph comprising approximately 10,000 nodes, computation time still ranged between 20 and 30 minutes. To further reduce computation time, we implemented parallelization, particularly in the stages of obtaining the SSSP and the accumulation phase, as outlined from lines 10 to 21 in Algorithm 1. This is why ‘*nodes*’ is an input of Algorithm 1.

Our parallelization approach employed multiprocessing in Python. We evenly distributed the total set of nodes across the processors and executed Algorithm 1 concurrently. The betweenness centrality values for each node and link were aggregated after computations on each processor, significantly reducing the total computation time to tens of seconds or a maximum of 2–3 minutes.

To evaluate the efficiency of our modified algorithm, we measured computation times across various graph sizes. Additionally, we measured computation time on a fixed-size graph with differing numbers of processors to assess the effect of parallelization. The experimental environment is summarized in Table 4 below.

**Table 4:** Experimental environment.

System	Configure
OS	Ubuntu 20.04.6 LTS
CPU	2 AMD EPYC 7502 32-Core Processor @ 2.5GHz (64 cores & 128 processors)
Memory	2TiB (32 64GiB DDR4 RAM @ 3.2GHz)

## Results and discussion

The research yielded two primary results: the formulation of a new betweenness centrality calculation algorithm that incorporates the node-port structure and the development of a visualization system designed to enhance on-site decision-making by highlighting identified structurally vulnerable links.

### Visualization system

**Fig 2:**
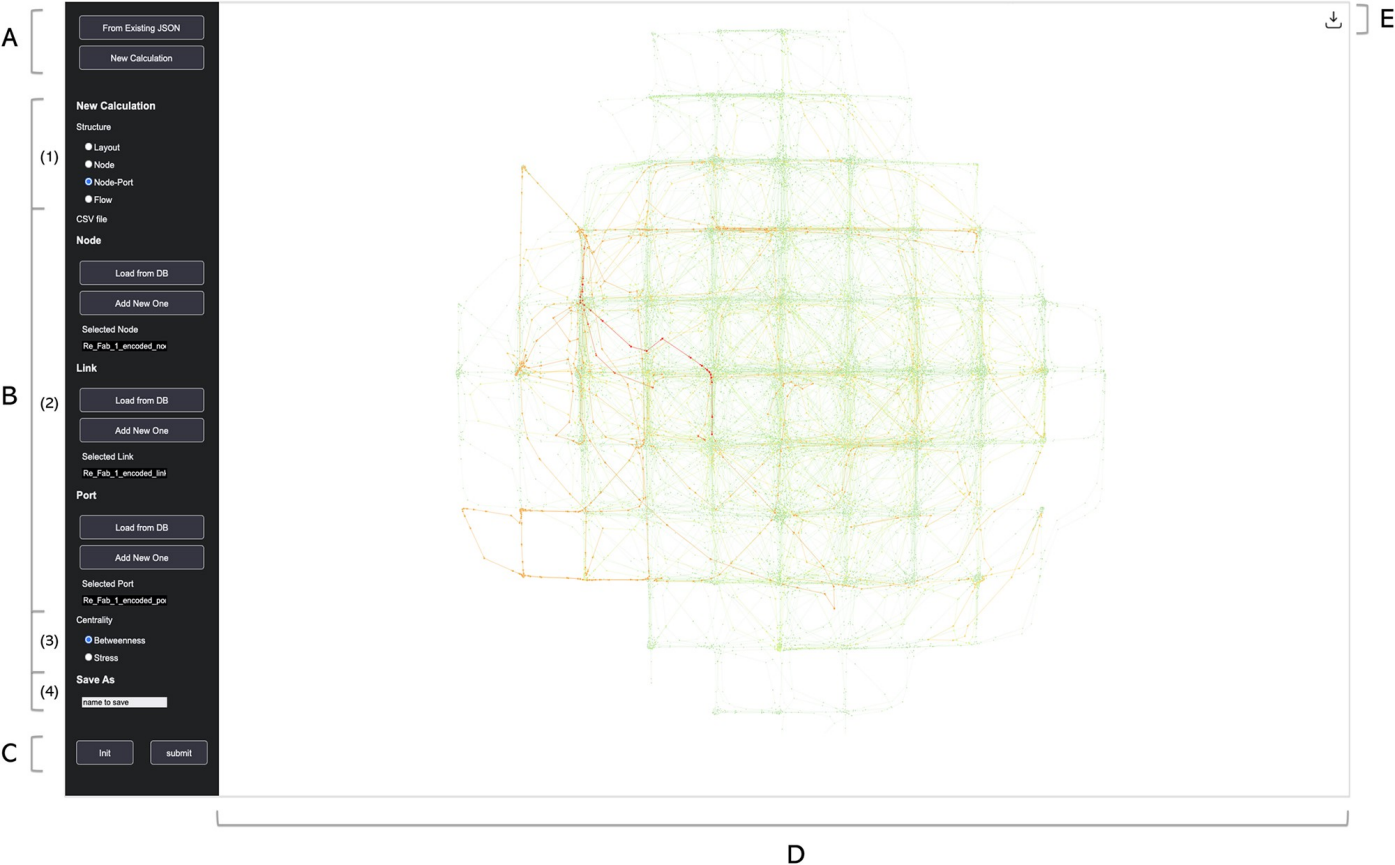
Visualization system overview. The A-C panels on the left sidebar with a black background form the ‘Analysis Panel,’ where users can load data and choose visualization options and the section D on the right with a white background is a space where the visualized network results can be viewed.

Fig 2 displays an overview of our visualization system. We divided our system into sections to describe the details of each part.

#### Analysis panel

The A-C parts on the left of Fig 2 form the ‘Analysis Panel,’ where users can select from various options to analyze the OHT railway network. The panel A allows users to either load pre-calculated results or calculate and visualize betweenness centrality through new calculations. It supports both immediate access to pre-calculated results and the processing of newly uploaded data. Users can click the ‘From Existing JSON’ button to load previously calculated results in JSON format or select ‘New Calculation’ and set specific parameters in panel B for new calculations. The panel B allows users to specify detailed parameters and load data for new network analysis. Section B-(1) provides four structural options: layout, node-only, node-port, and flow. ‘Layout’ shows the network structure alone, displaying the layout with node and link data. ‘Node’ and ‘Node-Port’ options calculate betweenness centrality for each node and link, with ‘Node’ excluding port data and ‘Node-Port’ using our modified Brandes algorithm to consider port data. The ‘Flow’ option visualizes the historical transport records of OHT, showing the total flow of OHT across nodes and links without additional calculations. The section B-(2) lets users load the necessary data for visualization. There are two primary methods: using the ‘Load from DB’ button to fetch data from the MongoDB database, or the ‘Add New One’ button to upload data from the user’s local computer. The available data includes node, link, port, and flow. Node and link are always required, while port and flow panels appear based on the ‘Node-Port’ and ‘Flow’ options chosen in B-(1). Users can select the type of centrality to calculate in the section B-(3). The ‘Centrality’ checkbox is enabled when either ‘Node’ or ‘Node-Port’ analysis is chosen in ‘B-(1).’ This system primarily calculates betweenness centrality to assess structural vulnerability but also offers stress centrality as a secondary measure. The section B-(4) is for saving the visualization results to the MongoDB database. Enter the desired name under ‘Save As,’ allowing the results to be easily reloaded later via the ‘From Existing JSON’ button in panel A. Lastly, the panel C includes a ‘submit’ button for visualizing the results after all options are set and an ‘init’ button to reset the system for a new analysis.

#### Visualization workspace

The section D of Fig 2 visualizes the OHT railway network after data is uploaded and the desired visualization options are selected in panels A-C. Betweenness and stress centrality are indicated by color-coded nodes and links, with size and thickness proportional to the centrality values. Higher values are represented by larger nodes and thicker links in red hues, while lower values are shown by smaller nodes and thinner links in green hues. The system provides several interactive features. Users can hover over nodes and links to see their centrality values, and zooming and panning enable focusing on specific network regions. Nodes and links can also be repositioned by dragging. Analytical results can be exported in CSV format via the download button in section ‘E’ at the top-right corner of the workspace. The CSV file provides the centrality results of nodes and links in descending order, making it easy for users to identify structurally vulnerable nodes and links.

### Comparative analysis

Identifying vulnerable links using betweenness centrality calculated by our newly modified algorithm demonstrated its significance by more accurately reflecting the operational conditions and traffic flows of the OHT railway network compared to the original Brandes algorithm.

**Fig 3:**
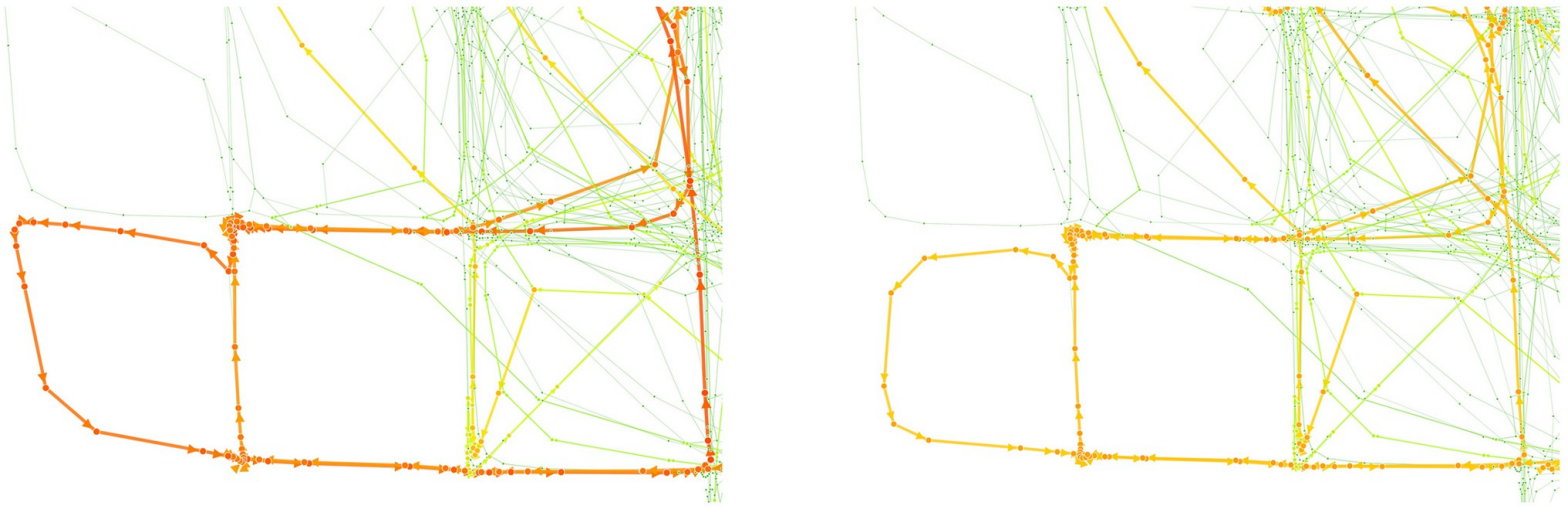
Comparison between the results of node-only and node-port structure. (left: node-only, right: node-port). The focused region is where bridge links are located. The betweenness centralities of bridge links are alleviated in the right figure.

Specifically, its effectiveness was evident in the ‘Bridge’ areas of the OHT railway network. Fig 3 shows the results of visualizing vulnerable links using betweenness centrality on different structure; the left side shows the result of traditional node-only structure with the Brandes algorithm, while the right side incorporates the node-port structure using our modified algorithm.

In the network layout, the lower left central section shifts from red in the node-only structure to orange and yellow hues in the node-port structure, indicating a decrease in betweenness centrality. Notably, these bridge areas, characterized by a minimal presence of ports, typically experience lower congestion levels in practical scenarios because they are used merely as passageways without OHT stopping points.

Our modified algorithm reflected the practical realities of the FAB more accurately by considering these conditions. It assigned lower vulnerability ratings to these bridge areas, avoiding the overestimation seen with the node-only structure that does not consider port presence. In a similar vein, our modified algorithm assessed higher vulnerability in areas with concentrated ports where OHTs frequently stop, reflecting the increased traffic since more shortest paths start from and lead to these ports. This inclusion of actual source and target ports in the algorithm better mirrors the real traffic conditions in the FAB.

**Fig 4:**
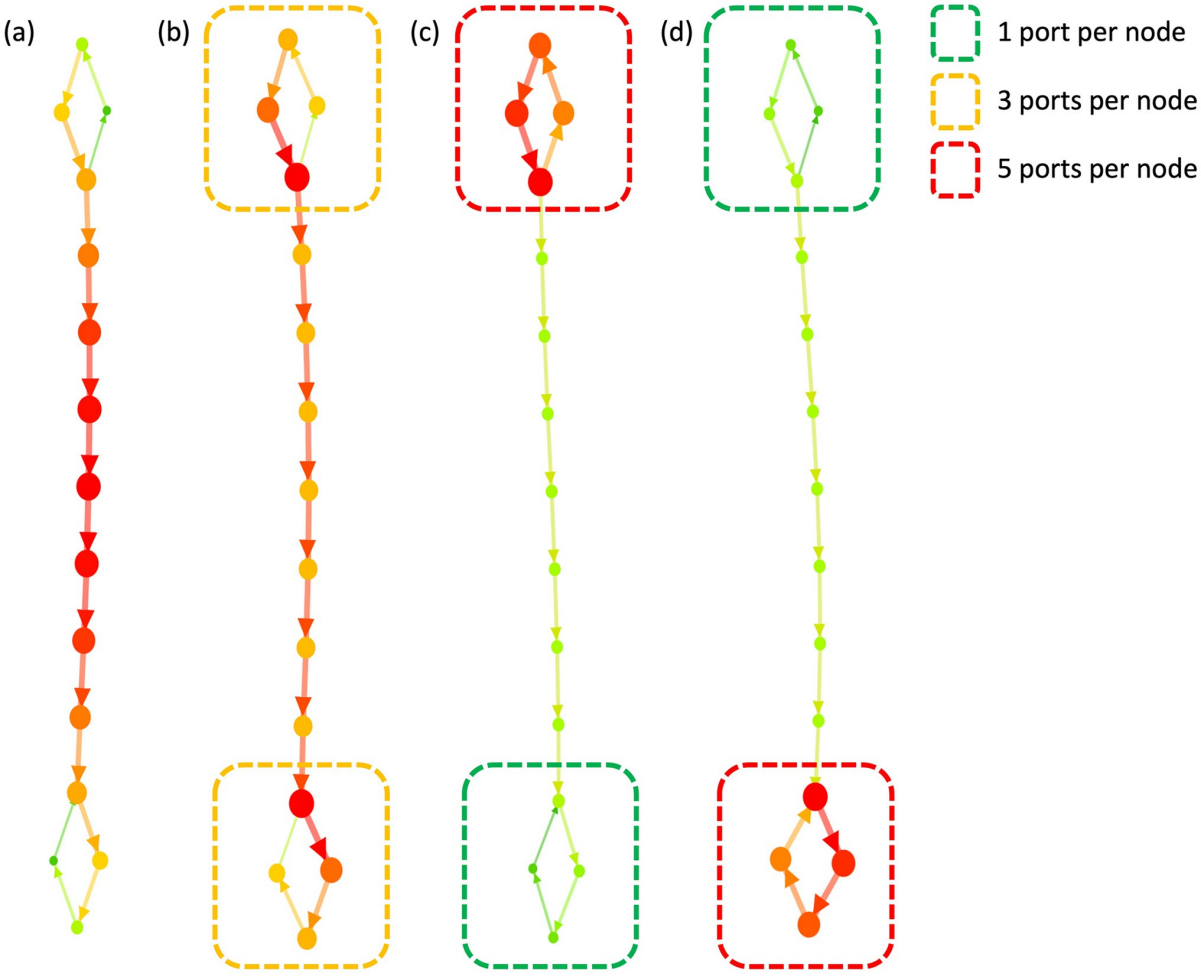
Effect of ports on betweenness centrality. An experiment on a simple bridge network structure was conducted, varying the number of ports per node in regions indicated by rectangles. (a) node-only, (b) equal port concentration at both ends. (c) higher port concentration at the upper end (d) higher port concentration at the lower end.

In order to clearly demonstrate the effect of port, we conducted an experiment varying the number of ports on a small network that includes a bridge, as depicted in Fig 4.

The outcome of Fig 4(a) represents the node-only result. We allocated ports to each individual node within the rectangular area. The color of the rectangle indicates the number of ports assigned to each node in the region: green for one port per node, orange for three ports per node, and red for five ports per node. The most vulnerable links are obviously the links located in the bridge region in Fig 4(a).

However, in Fig 4(b), an equal number of ports was assigned to each node at both ends of bridge. Despite this uniform assignment, the betweenness centralities at the bridge decreased, while those at the ends, where ports were assigned, increased.

This is because there are no ports on the bridge, meaning that the nodes on the bridge only serve as passing nodes and not as the source or target nodes. Furthermore, it can be observed that the region with a higher concentration of ports exhibited a higher level of betweenness centrality, as shown in Fig 4(c) and 4(d).

### Computational efficiency

#### Modified algorithm efficiency

To evaluate the efficiency of our modified algorithm, we conducted computational time tests on random graphs with node counts of 10, 100, 1000, 2500, and 5000. We opted not to measure time for the 10,000-node scenario due to the impractically long durations required by the original Brandes algorithm, which could extend to several hours. For each graph, the number of links was set to three times the number of nodes, and each node was assigned three ports. The original Brandes algorithm was conducted by treating ports as additional nodes.

**Fig 5:**
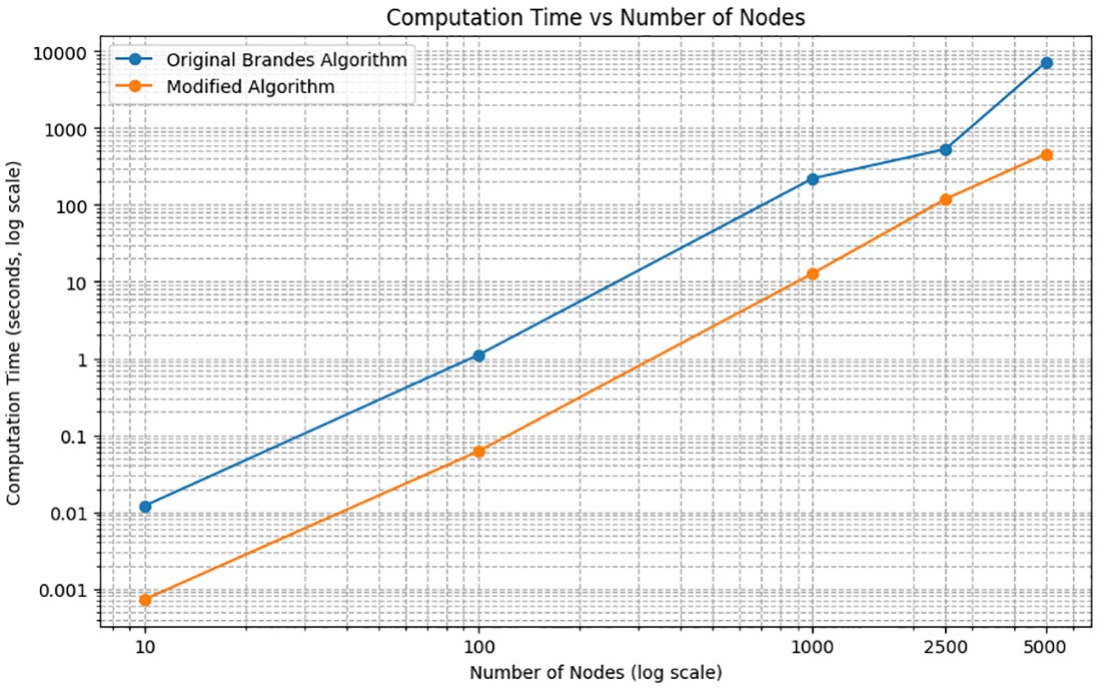
Graph of computation time vs number of nodes. The computation time was measured as the number of nodes increased, comparing the original Brandes algorithm with our modified algorithm. Our algorithm showed a significant improvement in computation time, with a 93.6% reduction for the 5000-node graph.

The results, presented in Fig 5, utilize a logarithmic scale on both the *x* and *y* axes to effectively display the wide range of graph sizes and computation times. The computation time for each algorithm increased with the graph size, ranging from 10 to 5000 nodes, as follows: the original Brandes algorithm recorded times of about 0.01, 1, 200, 600, and 7000 seconds; the modified algorithm showed times of about 0.001, 0.06, 10, 100, and 400 seconds, respectively, when plotted on a logarithmic scale. The results clearly showed a reduction in computation time by approximately an order of magnitude across all tested sizes. Specifically, for the 5000-node graph, it showed a 93.6% reduction in computation time compared to the original Brandes algorithm. Detailed data supporting these findings are shown in S1 Table in S1 File.

This reduction is expected as our modified Brandes algorithm only iterates over the original nodes, whereas the original Brandes algorithm iterates over additional port nodes, increasing the total node and link count. This modification substantially decreases computation time, irrespective of the number of supplementary nodes of ports. The algorithm’s applicability extends to various settings that feature a hierarchical structure similar to the node-port concept shown in Fig 1.

#### Parallelization efficiency

**Fig 6:**
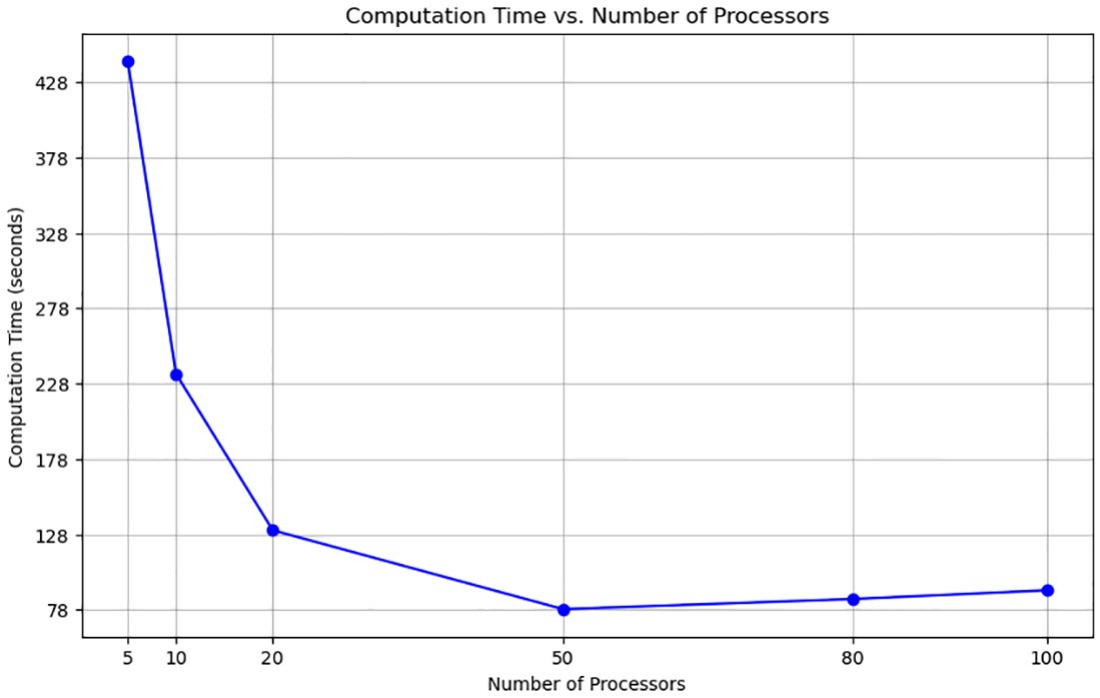
Graph of computation time vs number of processors. Parallelization efficiency was assessed by measuring computation time with a varying number of processors. A 5.7-times increase in computation speed was achieved with 50 processors.

While the modification of the Brandes algorithm successfully reduced computation time, its duration was still considerable for practical applications. To address this, we implemented parallelization using Python’s multiprocessing. We evaluated the algorithm’s performance on a graph comprising 10,000 nodes, maintaining the same link and port conditions as in previous experiments. The computation time was measured against varying numbers of processors: 5, 10, 20, 50, 80, and 100. The computation time significantly decreased, reaching the level of tens of seconds as shown in Fig 6. The axes are represented on a linear scale because the time range was narrow enough to be displayed linearly. The specific computation times for different processor counts are detailed in S2 Table in S1 File.

Notably, the computation time diminished up to the 50 processors, achieving an 82.4% reduction compared to using 5 processors, beyond which it began to slightly increase again. This rise in time can be attributed to the overhead associated with communication tasks, such as duplicating the entire graph object and port data across each processor’s memory, and the aggregation of results from individual processors. The aggregation process needs iteration a total number of times equal to the product of the number of processes and the combined count of nodes and links, and involves cumulatively summing the betweenness centrality results for each node and link. This factor is believed to be the primary cause of the increased computation time. Additional factors potentially influencing this trend may relate to the operational characteristics of multiprocessing in Python.

For graphs with node counts exceeding 10,000, utilizing a higher number of processors could further reduce computation time. However, considering that typical OHT railway networks approximate 10,000 nodes, we determined that setting the processor count to 50, which demonstrated optimal performance, was the most appropriate choice for our job.

Fast computation time may not be crucial when evaluating only a limited number of FAB layouts, as the results, once calculated, remain fixed. However, we have prioritized rapid computation for several reasons. Firstly, our system is a web-based visualization tool, and users expect immediate results. Secondly, the system can be employed to assess the robustness of virtual FAB layouts before actual construction. Rapid computation enable users to quickly test multiple configurations.

## Conclusion

This study successfully visualized the OHT railway network within a semiconductor FAB by integrating a force-directed layout algorithm with a grid-force layout algorithm. However, recognizing the exact nodes and links in our visualizations remains challenging due to the absence of precise coordinates. In future work, we may attempt to reconstruct the coordinates from distance or employ different layout algorithms to produce a clearer visualization of the FAB layout.

We modified the original Brandes algorithm to incorporate the node-port structure, thereby more accurately reflecting the dynamics of OHT movements within the FAB and enabling effective computation of the betweenness centrality. Through comparative analysis between the node-only and node-port structures, we validated that our approach yields more feasible and realistic results.

While we developed our modified algorithm to accommodate the node-port structure, it can also be extended to calculate the betweenness centrality of graphs with any hierarchical structure similar to the node-port structure. Additionally, we achieved a reduction in computation time by implementing parallelization using multiprocessing in the process of calculating betweenness centrality. This enhancement led to a more user-friendly computation duration, effectively balancing efficiency with the user’s needs.

Leveraging this system, domain experts in semiconductor FAB can identify structurally vulnerable links in the OHT railway network. Furthermore, the system serves as a preliminary tool for assesing the structural robustness of OHT railway network prior to their construction, offering insights that can guide design railway network structure.

Although we successfully identified structurally vulnerable links in OHT railway networks, future research could extend this work by incorporating betweenness centrality into more dynamic conditions, such as the deletion of links or the presence of moving OHTs on the railway.

## Supporting information

S1 File (PDF)
