# Supplementary material for: Visualization system to identify structurally vulnerable links in OHT railway network in semiconductor FAB using betweenness centrality
Source: PLoS One. 2024 Jul 12;19(7):e0307059. doi: 10.1371/journal.pone.0307059 (PMC11244790; doi:10.1371/journal.pone.0307059)
Supplement: S1 File — (PDF) [file pone.0307059.s001.pdf]

Supporting information

S1 Figure. Result using SMAT2022 layout data

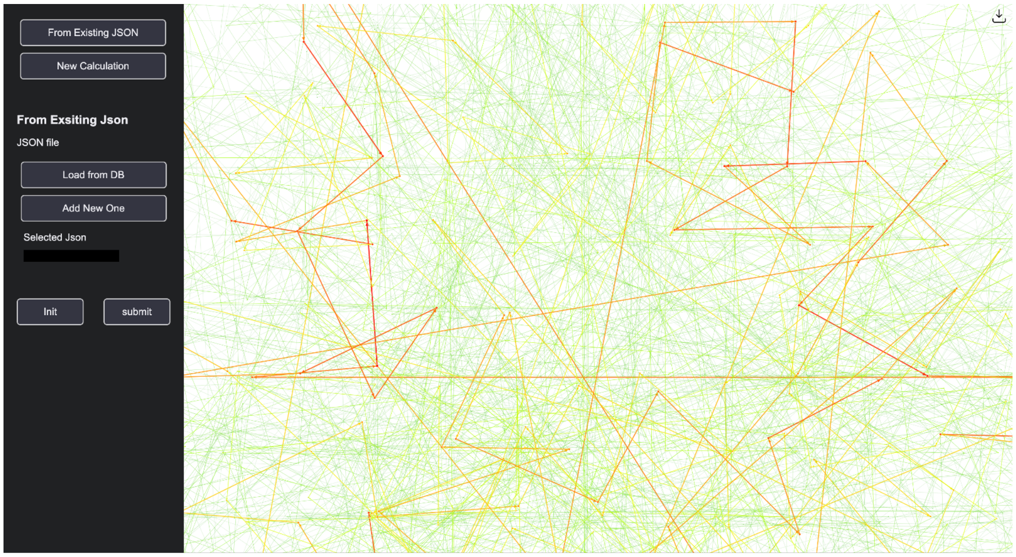

Fig 1. Visualization Result. Visualization result using the SMAT 2022 OHT railway layout open data.

S1 Appendix. Stress centrality

We employed stress centrality as a supplementary index for the vulnerability analysis. The definition of stress centrality is shown in Eq(1).

$$SC(v) = \sum_{s \neq v \neq t \in V} \sigma_{st}(v) \tag{1}$$

As you can see, stress centrality is just the sum of the number of all shortest paths that pass the node or link. We also considered node-only and node-port structure just like betweenness centrality.

stress centrality in node-only structure can be easily calculated just modifying the accumulation stage in Brandes algorithm. Since stress centrality doesn't consider the denominator term σ_{st}, the accumulation formula simply becomes like Eq(2).

$$\begin{aligned} \sum_{w: v \in P_s(w)} \sum_{t \in V} \sigma_{st}(v, \{v, w\}) &= \sum_{w: v \in P_s(w)} \left(\sigma_{sv} + \sum_{t \in V \setminus \{w\}} \frac{\sigma_{sv}}{\sigma_{sw}} \cdot \sigma_{st}(w) \right) \\ &= \sum_{w: v \in P_s(w)} \sigma_{sv} \cdot \left(1 + \frac{\sigma_{s\bullet}(w)}{\sigma_{sw}} \right) \end{aligned} \tag{2}$$

For node-port structure, we modified stress centrality as well as betweenness centrality case. Overall proof flow for stress centrality accumulation is same as betweenness centrality in node-port structure from Eq(3) to Eq(6). So we will omit detail description about the proof.

$$SC_p(v) = \sum_{s \neq v \neq t \in V} \sigma_{st}(v) p(s) p(t) = \sum_{s \neq v \neq t \in V} \sigma_{st,p}(v) \tag{3}$$

$$\sigma_{s\bullet,p}(v) = \sum_{t \in V} \sigma_{st,p}(v) = \sum_{t \in V} \sum_{w: v \in P_s(w)} \sigma_{st,p}(v, \{v, w\}) = \sum_{w: v \in P_s(w)} \sum_{t \in V} \sigma_{st,p}(v, \{v, w\}) \tag{4}$$

$$\sigma_{st,p}(v, \{v, w\}) = \begin{cases} \sigma_{sv} \cdot p(s) p(w) & \text{if } t = w \\ \\ \sigma_{sw}(v, \{v, w\}) \cdot \sigma_{wt} \cdot p(s) p(t) = \frac{\sigma_{sv}}{\sigma_{sw}} \cdot \sigma_{st}(w) \cdot p(s) p(t) & \text{if } t \neq w \end{cases} \tag{5}$$

$$\begin{aligned} \sum_{w: v \in P_s(w)} \sum_{t \in V} \sigma_{st,p}(v, \{v, w\}) &= \sum_{w: v \in P_s(w)} \left(\sigma_{sv} \cdot p(s) p(w) + \sum_{t \in V \setminus \{w\}} \frac{\sigma_{sv}}{\sigma_{sw}} \cdot \sigma_{st}(w) \cdot p(s) p(t) \right) \\ &= \sum_{w: v \in P_s(w)} \sigma_{sv} \cdot \left(p(s) p(w) + \frac{\sigma_{s\bullet,p}(w)}{\sigma_{sw}} \right) \end{aligned} \tag{6}$$

S1 Algorithm. Algorithm for stress centrality

Algorithm 1 Calculation of stress centrality

Input: $G = (V, E), weight, port, nodes$
Output: $SC\_node(v)$, $SC\_(e)$ for all $v \in V$ and $e \in E$

for $v \in V$ **do**
    $SC(v) \leftarrow 0$
end for
for $e \in V$ **do**
    $SC(e) \leftarrow 0$
end for
if $nodes$ = None **then**
    $nodes = V$
end if
for $s \in nodes$ **do**
    if $weight$ = None **then**
        $S, P, \sigma = SSSP(G, s)$
    else
        $S, P, \sigma = SSSP(G, s, weight)$
    end if
    if $port$ = None **then**
        $SC = SA(SC, S, P, sigma, s)$
    else
        $SC = SA_p(SC, S, P, sigma, s, port)$
    end if
end for
$SC\_node = \{\}$
for $s \in S(v)$ **do**
    pop SC_s from $SC(s)$
    $SC\_node(s) \leftarrow SC_s$
end for
$SC\_link = SC$
return $SC\_node(v)$, $SC\_link(e)$ for all $v \in V$ and $e \in E$

S2 Algorithm. Accumulation for stress centrality in node-port structure here $\delta(w)$ is used for $\sigma_{s\bullet}(w)$ to distinguish it from σ_{sw}

Algorithm 2 SA_p : Accumulate modified sigmas

Input: $SC, S, P, \sigma, s, port$
Output: $SC(x)$ for all $x \in V \cup E$

```
for v ∈ S do
    δ(v) ←0
    if v != s then
        sum_paths += port(v) ∗ σ(w)
    end if
end for
SC(s) += port(s) ∗ sum_paths
while S do
    pop w from S
    coeff = (port(s) ∗ port(w) + δ(w)/σ(w))
    for v ∈ P(w) do
        c = σ(v) ∗ coeff
        BC({v, w}) += c
        δ(v) += c
    end for
    if w! = s then
        SC(w) += δ(w) + port(s) ∗ port(w) ∗ σ(w)
    end if
end while
return SC(x) for all x ∈ V ∪ E
```

S1 Table. Data of computation time across the number of nodes in Fig 5

The number of nodes	Original Brandes algorithm (s)	Modified algorithm (s)	Efficiency gain (%)
10	1.21×10^{-2}	7.33×10^{-4}	93.96
100	1.12	6.22×10^{-2}	94.44
1000	2.22×10^{2}	1.28×10^{1}	94.21
2500	5.33×10^{2}	1.20×10^{2}	77.55
5000	7.27×10^{3}	4.62×10^{2}	93.64

S2 Table. Data of computation time across the number of processors in Fig 6

The number of processors	Computation time (s)
5	442.1
10	234.4
20	130.8
50	78.51
80	85.24
100	91.00
